# Checklist of bees (Hymenoptera: Apoidea) from small diversified vegetable farms in south-western Montana

**DOI:** 10.3897/BDJ.7.e30062

**Published:** 2019-01-28

**Authors:** Casey M. Delphia, Terry Griswold, Elizabeth G. Reese, Kevin M. O'Neill, Laura A. Burkle

**Affiliations:** 1 Departments of Ecology and Land Resources & Environmental Sciences, Montana State University, Bozeman, United States of America Departments of Ecology and Land Resources & Environmental Sciences, Montana State University Bozeman United States of America; 2 USDA-ARS Pollinating Insects Research Unit, Logan, United States of America USDA-ARS Pollinating Insects Research Unit Logan United States of America; 3 Department of Ecology, Montana State University, Bozeman, United States of America Department of Ecology, Montana State University Bozeman United States of America; 4 Department of Land Resources & Environmental Sciences, Montana State University, Bozeman, United States of America Department of Land Resources & Environmental Sciences, Montana State University Bozeman United States of America

**Keywords:** wild bees, native bees, pollinators, biodiversity, range expansion, farmlands, agroecosystems, Rocky Mountains, Intermountain West, wildflower strips, bee conservation

## Abstract

**Background:**

Over three years (2013-2015), we sampled bees using nets and bowl traps on four diversified vegetable farms in Gallatin County, Montana, USA, as part of a study evaluating the use of wildflower strips for supporting wild bees and crop pollination services on farmlands (Delphia et al. In prep). We document 202 species and morphospecies from 32 genera within five families, of which 25 species represent the first published state records for Montana. This study increases our overall understanding of the distribution of wild bee species associated with agroecosystems of the northern US Rockies, which is important for efforts aimed at conserving bee biodiversity and supporting sustainable crop pollination systems on farmlands.

**New information:**

We provide a species list of wild bees associated with diversified farmlands in Montana and increase the number of published bee species records in the state from 374 to at least 399. The list includes new distributional records for 25 wild bee species, including two species that represent considerable expansions of their known ranges, Lasioglossum (Dialictus) clematisellum (Cockerell 1904) with previously published records from New Mexico, Arizona, California and Utah and Melissodes (Eumelissodes) niveus Robertson 1895 which was reported to range from New York to Minnesota and Kansas, south to North Carolina, Alabama and Mississippi.

## Introduction

Native bees are important pollinators of wild and cultivated plants in natural habitats and agricultural systems (e.g. Losey and Vaughan 2006, Klein et al. 2007, Michener 2007, Garibaldi et al. 2013). In the United States and worldwide, however, bees and other pollinators are experiencing probable declines due to factors such as diseases, pesticides and habitat loss that can reduce floral resources and nesting sites (Biesmeijer et al. 2006, Potts et al. 2010, Cameron et al. 2011, Burkle et al. 2013, Goulson et al. 2015, Koh et al. 2016). However, because we lack a baseline understanding of the bee species that occur in many parts of the US, particularly in certain western regions of the country, concerns regarding the status and trends of wild bees cannot be accurately assessed.

Montana’s bee fauna is one of the least-studied amongst US states (but see Kuhlman and Burrows 2017, Dolan et al. 2017, Reese et al. 2018); of the few studies that have been conducted in Montana, even fewer have examined wild bees in agricultural systems. Documenting the diversity of bees on farmlands is important for identifying potential crop pollinators, for gauging the potential of farmland habitats to support overall bee diversity on and around farms and for guiding bee conservation measures (Burkle et al. 2017). In addition, the spine of North America, which is the Rocky Mountains, extends from British Columbia to New Mexico and runs through western Montana. This major geographic barrier separates east and west biotas and provides a habitat for alpine/boreal species. Therefore, understanding wild bee distributions in Montana is biogeographically important because its regional species pool likely includes many bee species with typically eastern, western, arctic and southern US ranges that overlap within the state (Gibbs 2010, Koch et al. 2012, Williams et al. 2014, Dolan et al. 2017). A 2017 study, for example, documented 28 bumble bee species in the state, several of which were previously considered to have purely eastern or western North American ranges (Dolan et al. 2017). Another recent survey documented 34 bee species as new state records from just one of Montana’s 56 counties (Kuhlman and Burrows 2017). The results from these studies, coupled with Montana’s large size (381,000 km^2^) and diverse ecosystems, many of which are difficult to access, suggest there is much to learn about the wild bee fauna in this region.

We report a checklist of bee species from a three-year study surveying the bee community on diversified vegetable farms in south-western Montana. This is the second study (along with Adhikari et al. In prep) in the state to survey wild bees in agroecosystems, though the habitats (Greater Yellowstone Ecosystem versus Northern Great Plains), type of farming systems (diversified versus highly-simplified) and crops (pollinator-dependent versus wind-pollinated) differed extensively. This work contributes to our long-term goal of creating a comprehensive bee species list for the state.

## Materials and methods

**Study Sites**

**Figure 1.**
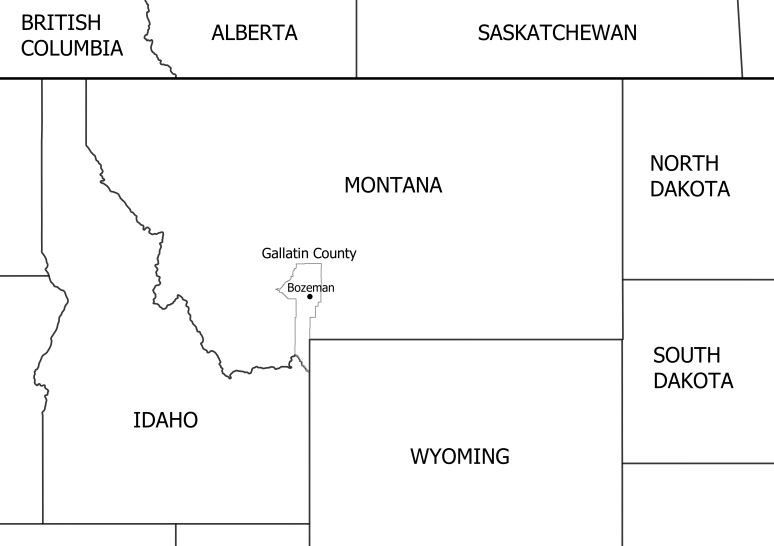
Map of Montana and surrounding Canadian provinces and US states with Gallatin County boundary and city of Bozeman marked. All sampling took place within a 24 km radius of Bozeman, MT.

This research was conducted at four diversified farms located in south-western Montana USA within a 24 km radius of Bozeman (45.6769°N; 111.0429°W) in Gallatin County (Fig. 1, Table 1). The farms we surveyed are within the eastern end of the broad Gallatin Valley, which is surrounded by five mountain ranges, two of which are nearby: the Bridger Mountains to the northeast and the Gallatin Range to the south. Each farm had approximately 3-7 acres in cultivation each year and grew a variety of crops, including squashes and pumpkins (*Cucurbita pepo* L.), tomatoes (*Solanum lysopersicum* L.), cucumbers (*Cucumis sativus* L.) and strawberries (*Fragaria x ananassa* Duchesne), marketed locally through Community Supported Agriculture (CSA) programmes, farmers’ markets, food co-ops and restaurants; two of the farms were certified organic and two followed organic or sustainable practices. Elevations of the farms ranged from 1350-1511 m above sea level. The mean annual precipitation in the area is 469 mm, the mean annual daily high temperature is 12.89°C and the mean annual low temperature is -0.44°C (Western Regional Climate Center 2018; Suppl. material 1). As we were interested in evaluating wildflower strips for supporting bees and crop pollination, our experimental design included planting wildflowers and experimental crop strips from which we sampled bees.

**Collection Methods**

We collected bees on each farm from May-September in 2013-2015; all sampling took place on calm, sunny days between 0900 and 1700 h MDT. We net-collected bees visiting the reproductive parts of flowers of species blooming in: 1) the established wildflower strips (*Campanula rotundifolia* L., *Erigeron speciosus* (Lindl.) DC., *Gaillardia aristata* Pursh, *Geranium viscosissimum* Fisch. & C.A. Mey. Ex C.A. Mey., *Helianthus maximiliani* Schrad., *Heterotheca villosa* (Pursh) Shinners, *Monarda fistulosa* L., *Penstemon confertus* Douglas ex Lindl. and *Phacelia hastata* Douglas ex Lehm.) during weekly timed observations in 2014 and 2015; 2) crops (acorn winter squash, *Cucurbita pepo* and sunflower, *Helianthus annuus* L.) during timed observations every other week in 2013 or weekly in 2014 and 2015; and 3) other prolific bloomers, which included primarily agricultural weeds (e.g. common tansy, *Tanacetum vulgare* L. and Canada thistle, *Cirsium arvense* (L.) Scop.), during timed observations once or twice a month, depending on the amount of surrounding vegetation and time availability. We collected for a total of ca. 23 hours in 2013, 104 hours in 2014 and 85 hours in 2015. In all three years, the same experienced netter (15 years) was paired with a less-experienced netter (≤1 year) for bee collections with both contributing equal amounts of time to collecting; in 2015 the same netter from 2014 assisted with bee collections. Bees were freeze-killed, pinned, and labelled. We also collected bees weekly using yellow, 350-ml Solo bowls filled with soapy water. Six bowls were deployed approximately 6 m apart along each of four, 33-m linear transects (24 bowls per farm) located at different distances from the wildflower strips and left out for approximately six hours during the height of bee activity. Samples were collected into 70% EtOH and later removed from the alcohol, washed, blow-dried, pinned and labelled. We used EstimateS (Colwell 2013) to generate a Chao1 species richness predictor to estimate the “true” number of species present.

**Species Identification**

We identified bees to the lowest taxonomic level possible using published keys (Table 2) following the classifications of Michener (2007); specimens were identified to subspecies only when they could be accurately assigned. We used reference specimens from the US National Pollinating Insect Collection to identify a comprehensive subset of the bees collected in this study; these were then taken back to the O’Neill and Burkle Laboratories to use as reference specimens for species identifications and verification of the remaining material.

For genera where no taxonomic literature was available for species-level identifications, we grouped bees that appeared morphologically distinct into morphospecies. We assigned each morphospecies a unique number and the letter “F” for females and “M” for males. However, because we could not reliably associate male and female morphospecies as a single “species” and to avoid inflating species numbers, we included only female morphospecies in this checklist. Species names with *aff.* (‘has affinity with’) are also treated as morphospecies.

As females of *Agapostemon angelicus* Cockerell and *Agapostemon texanus* Cresson are indistinguishable from one another (Roberts 1972), they have been included with the confirmed males for species counts. Similarly, we could not distinguish amongst females of *Hylaeus mesillae* (Cockerell) and *Hylaeus rudbeckiae* (Cockerell and Casad) and they have been included with the males for species counts. For the Chao1 analysis, we have distributed the numbers of females of *A. angelicus/texanus* and *H. mesillae/rudbeckiae* according to the proportions of confirmed males of each species for each of the two genera.

Due to a paucity of regional keys and an inability to discern any distinctive characters amongst individuals, except for one morphospecies, bees in the genus *Sphecodes* were only identified to genus. Similarly, *Lasioglossum* of the subgenus *Evylaeus* were identified only to subgenus. Due to time and resource constraints, a randomly-chosen subsample of 66 of the 4,173 Lasioglossum (Dialictus) collected in this study were identified to species.

Voucher specimens will be deposited in the Montana Entomology Collection (MTEC) at Montana State University, Bozeman, MT USA.

**Range**

To determine whether a species was a new state record, we compared our checklist with other published checklists and literature focused on Montana fauna (O’Neill and Seibert 1996, Fultz 2005, Pearce et al. 2012, Kuhlman and Burrows 2017, Dolan et al. 2017, Reese et al. 2018), as well as a recent unpublished study (Adhikari et al. In prep). These comparisons revealed a subset of species unique to our study; to ensure that these were first records for the state, we reviewed each of these species in the Catalog of Hymenoptera in America north of Mexico (Hurd 1979) for additional Montana records. Where the catalogue included a record from Montana, we did not conduct further searches for specific localities since our goal was to discover new records for the state. For those not listed as present in the catalogue, we used it to guide further literature searches. We then used published primary literature (see Literature Cited below in checklist notes) to further search for Montana records and determine species ranges. Our search revealed 25 unpublished state records. We also searched  DiscoverLife.org; it revealed unpublished records with specific locality information for 5 of the 25 species (Ascher and Pickering 2018; Suppl. material 2), which are indicated below in the checklist notes. For each of the 25 new state records, we also provide information on the closest records reported within the same literature examined for Montana records (see checklist notes).

## Checklists

### Checklist

#### Andrenidae

#### Andreninae

#### Andrenini

#### Andrena (Andrena) thaspii

Graenicher 1903

##### Notes

Table 1: Sites 1-3.

#### Andrena (Andrena) topazana

Cockerell 1906

##### Notes

Table 1: Sites 1, 3.

#### Andrena (Callandrena) helianthi

Robertson 1891

##### Notes

Table 1: Sites 1-4.

#### Andrena (Cnemidandrena) costillensis

Viereck & Cockerell 1914

##### Notes

New species record for Montana (Donovan 1977; Table 1: Sites 2, 3). Unpublished record on DiscoverLife (Suppl. material 2). The closest records reported in Donovan (1977) for this species are from neighbouring states Idaho and Wyoming.

#### Andrena (Diandrena) nothocalaidis

(Cockerell 1905)

##### Notes

Table 1: Sites 2, 3.

#### Andrena (Euandrena) astragali

Viereck & Cockerell 1914

##### Notes

Table 1: Sites 2-4.

#### Andrena (Euandrena) nigrocaerulea

Cockerell 1897

##### Notes

Table 1: Sites 1-4.

#### Andrena (Holandrena) cressonii infasciata

Robertson 1891

##### Notes

Table 1: Sites 1-4.

#### Andrena (Melandrena) nivalis

Smith 1853

##### Notes

Table 1: Sites 2, 3.

#### Andrena (Micrandrena) microchlora

Cockerell 1922

##### Notes

Table 1: Sites 2, 3.

#### Andrena (Plastandrena) prunorum

Cockerell 1896

##### Notes

Table 1: Sites 1-4.

#### Andrena (Scaphandrena) scurra

Viereck 1904

##### Notes

Table 1: Site 1.

#### Andrena (Simandrena) nasonii

Robertson 1895

##### Notes

New species record for Montana (LaBerge 1989; Table 1: Sites 1, 2). The closest record reported in LaBerge (1989) for this species is from neighbouring state North Dakota.

#### Andrena (Simandrena) pallidifovea

(Viereck 1904)

##### Notes

Table 1: Sites 2, 3.

#### Andrena (Thysandrena) candida

Smith 1879

##### Notes

Table 1: Sites 2-4.

#### Andrena (Thysandrena) medionitens

Cockerell 1902

##### Notes

Table 1: Sites 1-4.

#### Andrena (Thysandrena) vierecki

Cockerell 1904

##### Notes

Table 1: Site 2.

#### Andrena (Trachandrena) hippotes

Robertson 1895

##### Notes

Table 1: Site 2.

#### Andrena (Trachandrena) miranda

Smith 1879

##### Notes

Table 1: Sites 2, 3.

#### Andrena (Tylandrena) aff. wilmattae

Cockerell 1906

##### Notes

Table 1: Site 1.

#### Andrena sp. F1

##### Notes

Table 1: Sites 2, 3.

#### Andrena sp. F2

##### Notes

Table 1: Sites 2-4.

#### Andrena sp. F3

##### Notes

Table 1: Site 3.

#### Panurginae

#### Calliopsini

#### Calliopsis (Calliopsima) chlorops

Cockerell 1899

##### Notes

New species record for Montana (Shinn 1967; Table 1: Sites 1, 3). The closest records reported in Shinn (1967) for this species are from neighbouring states Idaho and Wyoming.

#### Calliopsis (Calliopsima) coloradensis

Cresson 1878

##### Notes

Table 1: Site 3.

#### Calliopsis (Calliopsis) andreniformis

Smith 1853

##### Notes

Table 1: Sites 2-4.

#### Calliopsis (Nomadopsis) personata

Cockerell 1897

##### Notes

New species record for Montana (Timberlake 1952, Rozen 1958; Table 1: Site 1). The closest records reported in Timberlake (1952) and Rozen (1958) for this species are from neighbouring states Idaho and Wyoming.

#### Panurgini

#### Panurginus atriceps

(Cresson 1878)

##### Notes

Table 1: Sites 1-4.

#### Panurginus sp. F1

##### Notes

Table 1: Sites 1-4.

#### Perditini

#### Perdita (Cockerellia) lingualis

Cockerell 1896

##### Notes

New species record for Montana (Timberlake 1960; Table 1: Site 1). The closest records reported in Timberlake (1960) for this species are from Colorado, Nebraska and Utah. Mayer et al. (2000) reported this species from south-eastern Washington.

#### Perdita (Perdita) fallax

Cockerell 1896

##### Notes

Table 1: Site 3.

#### Perdita (Perdita) salicis

Cockerell 1896

##### Notes

New species record for Montana (Timberlake 1964; Table 1: Sites 1, 4). The closest record reported in Timberlake (1964) for this species is from neighbouring state Idaho.

#### Perdita (Pygoperdita) wyomingensis segona

Timberlake 1956

##### Notes

Table 1: Sites 2, 3.

#### Protandrenini

#### Protandrena (Pterosarus) innuptus

(Cockerell 1896)

##### Notes

Table 1: Sites 1-3.

#### Protandrena (Pterosarus) irregularis

(Cockerell 1922)

##### Notes

New species record for Montana (Cockerell 1922; Table 1: Site 1). Unpublished record on DiscoverLife (Suppl. material 2). The closest record reported in Cockerell (1922) and Hurd (1979) for this species is from Colorado.

#### Protandrena (Pterosarus) piercei

(Crawford 1903)

##### Notes

New species record for Montana (Cockerell 1922; Table 1: Site 1). The closest records reported in Hurd (1979) for this species are from neighbouring Canadian province Alberta and from neighbouring US state North Dakota.

#### Apidae

#### Apinae

#### Anthophorini

#### Anthophora (Clisodon) terminalis

Cresson 1869

##### Notes

Table 1: Sites 1-4.

#### Anthophora (Melea) bomboides

Kirby 1837

##### Notes

Table 1: Site 1.

#### Anthophora (Mystacanthophora) urbana

Cresson 1878

##### Notes

Table 1: Sites 1, 3, 4.

#### Apini

#### Apis (Apis) mellifera

Linnaeus 1758

##### Ecological interactions

###### Native status

Non-native to North America.

##### Notes

Table 1: Sites 1-4.

#### Bombini

#### Bombus (Bombias) nevadensis

Cresson 1874

##### Notes

Table 1: Sites 1-4.

#### Bombus (Bombus) occidentalis

Greene 1858

##### Notes

Table 1: Sites 1-3.

#### Bombus (Cullumanobombus) griseocollis

(De Geer 1773)

##### Notes

Table 1: Site 1.

#### Bombus (Cullumanobombus) rufocinctus

Cresson 1863

##### Notes

Table 1: Sites 1-4.

#### Bombus (Psithyrus) insularis

(Smith 1861)

##### Notes

Table 1: Sites 1-4.

#### Bombus (Pyrobombus) bifarius

Cresson 1878

##### Notes

Table 1: Sites 1-4.

#### Bombus (Pyrobombus) centralis

Cresson 1864

##### Notes

Table 1: Sites 1-4.

#### Bombus (Pyrobombus) flavifrons

Cresson 1863

##### Notes

Table 1: Sites 2, 4.

#### Bombus (Pyrobombus) huntii

Greene 1860

##### Notes

Table 1: Sites 1-4.

#### Bombus (Pyrobombus) mixtus

Cresson 1878

##### Notes

Table 1: Sites 1-4.

#### Bombus (Pyrobombus) sylvicola

Kirby 1837

##### Notes

Table 1: Site 4.

#### Bombus (Subterraneobombus) appositus

Cresson 1878

##### Notes

Table 1: Sites 1-3.

#### Bombus (Thoracobombus) fervidus

(Fabricius 1798)

##### Notes

Table 1: Site 1.

#### Emphorini

#### Diadasia (Coquillettapis) diminuta

(Cresson 1878)

##### Notes

Table 1: Sites 1, 3, 4.

#### Eucerini

#### Melissodes (Callimelissodes) lupinus

Cresson 1878

##### Notes

Table 1: Sites 1, 4.

#### Melissodes (Callimelissodes) metenua

Cockerell 1924

##### Notes

New species record for Montana (LaBerge 1961; Table 1: Site 3). The closest records reported in LaBerge (1961) and Hurd (1979) for this species are from neighbouring states Idaho and Wyoming.

#### Melissodes (Eumelissodes) agilis

Cresson 1878

##### Notes

Table 1: Sites 1-4.

#### Melissodes (Eumelissodes) confusus

Cresson 1878

##### Notes

Table 1: Site 4.

#### Melissodes (Eumelissodes) coreopsis

Robertson 1905

##### Notes

Table 1: Sites 1-4.

#### Melissodes (Eumelissodes) hymenoxidis

Cockerell 1906

##### Notes

Table 1: Site 4.

#### Melissodes (Eumelissodes) menuachus

Cresson 1868

##### Notes

Table 1: Site 1.

#### Melissodes (Eumelissodes) microstictus

Cockerell 1905

##### Notes

Table 1: Sites 1-4.

#### Melissodes (Eumelissodes) niveus

Robertson 1895

##### Notes

New species record for Montana (LaBerge 1961; Table 1: Site 1). The closest records reported in LaBerge (1961) for this species are from Minnesota and Nebraska.

#### Melissodes (Eumelissodes) pallidisignatus

Cockerell 1905

##### Notes

Table 1: Site 1.

#### Melissodes (Eumelissodes) perlusus

Cockerell 1925

##### Notes

New species record for Montana (LaBerge 1961; Table 1: Site 1). The closest records reported in LaBerge (1961) for this species are from neighbouring Canadian province Alberta and from neighbouring US states North Dakota and Wyoming.

#### Melissodes (Melissodes) communis

Cresson 1878

##### Notes

New species record for Montana (LaBerge 1956b; Table 1: Sites 3, 4). The closest records reported in LaBerge (1956b) for this species are from neighbouring Canadian provinces British Columbia and Alberta and from neighbouring US states Idaho, North Dakota, South Dakota and Wyoming.

#### Melectini

#### Xeromelecta (Melectomorpha) californica

(Cresson 1878)

##### Notes

Table 1: Site 1.

#### Nomadinae

#### Epeolini

#### Epeolus sp. F1

##### Notes

Table 1: Site 3.

#### Triepeolus paenepectoralis

Viereck 1905

##### Notes

Table 1: Sites 1, 3, 4.

#### Triepeolus sp. F1

##### Notes

Table 1: Sites 1, 3, 4.

#### Triepeolus sp. F2

##### Notes

Table 1: Site 4.

#### Nomadini

#### Nomada ruficornis group sp. F1

##### Notes

Bidentate; Table 1: Site 2.

#### Nomada ruficornis group sp. F2

##### Notes

Unidentate; Table 1: Sites 1, 4.

#### Nomada ruficornis group sp. F3

##### Notes

Unidentate; Table 1: Site 2.

#### Nomada ruficornis group sp. F4

##### Notes

Bidentate; Table 1: Site 2.

#### Nomada ruficornis group sp. F5

##### Notes

Bidentate; Table 1: Sites 2, 3.

#### Nomada ruficornis group sp. F6

##### Notes

Bidentate; Table 1: Sites 2-4.

#### Nomada ruficornis group sp. F7

##### Notes

Unidentate; Table 1: Sites 1, 2, 4.

#### Nomada ruficornis group sp. F8

##### Notes

Unidentate; Table 1: Sites 1, 4.

#### Nomada ruficornis group sp. F9

##### Notes

Unidentate; Table 1: Site 1.

#### Nomada ruficornis group sp. F10

##### Notes

Unidentate; Table 1: Sites 2, 4.

#### Nomada ruficornis group sp. F11

##### Notes

Unidentate; Table 1: Sites 2, 3.

#### Xylocopinae

#### Ceratinini

#### Ceratina (Zadontomerus) acantha

Provancher 1895

##### Notes

Table 1: Site 3.

#### Ceratina (Zadontomerus) nanula

Cockerell 1897

##### Notes

Table 1: Sites 2, 3.

#### Ceratina (Zadontomerus) neomexicana

Cockerell 1901

##### Notes

Table 1: Sites 2-4.

#### Colletidae

#### Colletinae

#### Colletini

#### Colletes aff. phaceliae

Cockerell 1906

##### Notes

Table 1: Site 4.

#### Colletes consors pascoensis

Cockerell 1898

##### Notes

Table 1: Site 4.

#### Colletes fulgidus fulgidus

Swenk 1904

##### Notes

Table 1: Sites 1-4.

#### Colletes kincaidii

Cockerell 1898

##### Notes

Table 1: Site 2.

#### Colletes lutzi lutzi

Timberlake 1943

##### Notes

Table 1: Site 1.

#### Colletes nigrifrons

Titus 1900

##### Notes

Table 1: Site 2.

#### Colletes phaceliae

Cockerell 1906

##### Notes

Table 1: Sites 2-4.

#### Hylaeinae

#### Hylaeus (Cephalylaeus) basalis

(Smith 1853)

##### Notes

Table 1: Site 3.

#### Hylaeus (Hylaeus) annulatus

(Linnaeus 1758)

##### Notes

Table 1: Sites 2, 3.

#### Hylaeus (Hylaeus) conspicuus

(Metz 1911)

##### Notes

Table 1: Site 3.

#### Hylaeus (Hylaeus) leptocephalus

(Morawitz 1871)

##### Ecological interactions

###### Native status

Non-native to North America.

##### Notes

Table 1: Site 1.

#### Hylaeus (Hylaeus) mesillae

(Cockerell 1896)

##### Notes

Table 1: Sites 2-4.

#### Hylaeus (Hylaeus) rudbeckiae

(Cockerell and Casad 1895)

##### Notes

Table 1: Sites 1-4.

#### Hylaeus (Hylaeus) verticalis

(Cresson 1869)

##### Notes

Table 1: Sites 2, 3.

#### Hylaeus (Paraprosopis) coloradensis

(Cockerell 1896)

##### Notes

Table 1: Sites 2-4.

#### Hylaeus (Paraprosopis) wootoni

(Cockerell 1896)

##### Notes

Table 1: Sites 1-4.

#### Hylaeus (Prosopis) affinis

(Smith 1853)

##### Notes

New species record for Montana (Metz 1911, Snelling 1966a; Table 1: Sites 2-4). The closest records reported in Snelling (1966a) for this species are from neighbouring Canadian provinces British Columbia and Saskatchewan and from neighbouring US state Idaho.

#### Hylaeus (Prosopis) modestus

Say 1837

##### Notes

Table 1: Sites 1-3.

#### Halictidae

#### Halictinae

#### Augochlorini

#### Augochlorella aurata

(Smith 1853)

##### Notes

Table 1: Sites 2, 3.

#### Halictini

#### Agapostemon (Agapostemon) angelicus

Cockerell 1924

##### Notes

Table 1: Sites 1, 3, 4.

#### Agapostemon (Agapostemon) femoratus

Crawford 1901

##### Notes

Table 1: Site 1.

#### Agapostemon (Agapostemon) texanus

Cresson 1872

##### Notes

Table 1: Site 1.

#### Agapostemon (Agapostemon) virescens

(Fabricius 1775)

##### Notes

Table 1: Sites 1-4.

#### Halictus (Odontalictus) ligatus

Say 1837

##### Notes

Table 1: Sites 1-4.

#### Halictus (Protohalictus) rubicundus

(Christ 1791)

##### Notes

Table 1: Sites 1-4.

#### Halictus (Seladonia) confusus

Smith 1853

##### Notes

Table 1: Sites 1-4.

#### Halictus (Seladonia) tripartitus

Cockerell 1895

##### Notes

Table 1: Sites 1-4.

#### Lasioglossum (Dialictus) aff. admirandum

(Sandhouse 1924)

##### Notes

Table 1: Sites 2, 4.

#### Lasioglossum (Dialictus) clematisellum

(Cockerell 1904)

##### Notes

New species record for Montana (Sandhouse 1924; Table 1: Site 1). The closest record reported in Hurd (1979) for this species is from Utah.

#### Lasioglossum (Dialictus) cressonii

(Robertson 1890)

##### Notes

New species record for Montana (Sandhouse 1924, Gibbs 2010; Table 1: Site 4). Unpublished record on DiscoverLife (Suppl. material 2). The closest records reported in Gibbs (2010) for this species are from neighbouring Canadian provinces British Columbia and Alberta.

#### Lasioglossum (Dialictus) laevissimum

(Smith 1853)

##### Notes

New species record for Montana (Sandhouse 1924, Gibbs 2010; Table 1: Sites 1-4). Unpublished record on DiscoverLife Suppl. material 2). The closest records reported in Gibbs (2010) for this species are from neighbouring Canadian provinces British Columbia, Alberta and Saskatchewan.

#### Lasioglossum (Dialictus) lilliputense

Gibbs 2010

##### Notes

Table 1: Sites 2-4.

#### Lasioglossum (Dialictus) lineatulum

(Crawford 1906)

##### Notes

New species record for Montana (Gibbs 2010; Table 1: Site 2). The closest records reported in Gibbs (2010) for this species are from neighbouring Canadian provinces Alberta and Saskatchewan.

#### Lasioglossum (Dialictus) occidentale

(Crawford 1902)

##### Notes

Table 1: Sites 1-4.

#### Lasioglossum (Dialictus) semicaeruleum

(Cockerell 1895)

##### Notes

Table 1: Sites 1, 3.

#### Lasioglossum (Dialictus) succinipenne

(Ellis 1913)

##### Notes

Table 1: Site 2.

#### Lasioglossum (Dialictus) versans

(Lovell 1905)

##### Notes

New species record for Montana (Sandhouse 1924, Gibbs 2010; Table 1: Site 3). The closest record reported in Gibbs (2010) for this species is from neighbouring Canadian province Alberta.

#### Lasioglossum (Dialictus) zephyrum

(Smith 1853)

##### Notes

Table 1: Site 1.

#### Lasioglossum (Dialictus) spp.

##### Notes

Table 1: Sites 1-4.

#### Lasioglossum (Evylaeus) spp.

##### Notes

Table 1: Site 1.

#### Lasioglossum (Hemihalictus) ovaliceps

(Cockerell, 1898)

##### Notes

Table 1: Site 3.

#### Lasioglossum (Lasioglossum) egregium

(Vachal 1904)

##### Notes

Table 1: Site 2.

#### Lasioglossum (Lasioglossum) mellipes

(Crawford 1907)

##### Notes

New species record for Montana (McGinley 1986; Table 1: Site 4). The closest records reported in McGinley (1986) for this species are from neighbouring Canadian province British Columbia and from neighbouring US state Idaho.

#### Lasioglossum (Lasioglossum) paraforbesii

McGinley 1986

##### Notes

Table 1: Site 1.

#### Lasioglossum (Lasioglossum) sisymbrii

(Cockerell 1895)

##### Notes

Table 1: Site 2.

#### Lasioglossum (Lasioglossum) titusi

(Crawford 1902)

##### Notes

Table 1: Sites 2, 3.

#### Lasioglossum (Leuchalictus) zonulum

(Smith 1848)

##### Notes

Table 1: Sites 1, 4.

#### Lasioglossum (Sphecodogastra) lusorium

(Cresson 1872)

##### Notes

Table 1: Site 1.

#### Sphecodes sp. F1

##### Notes

Table 1: Sites 2, 4.

#### Sphecodes spp.

##### Notes

Table 1: Sites 1-4.

#### Rophitinae

#### Dufourea marginata

(Cresson 1878)

##### Notes

New species record for Montana (Michener 1951; Table 1: Sites 1, 4). The closest records reported in Hurd (1979) for this species are from neighbouring Canadian province Alberta and from neighbouring state Wyoming.

#### Dufourea maura

(Cresson 1878)

##### Notes

Table 1: Sites 2, 3.

#### Dufourea trochantera

Bohart 1948

##### Notes

Table 1: Site 4.

#### Megachilidae

#### Megachilinae

#### Anthidiini

#### Anthidium (Anthidium) mormonum

Cresson 1878

##### Notes

Table 1: Sites 1, 3.

#### Anthidium (Anthidium) tenuiflorae

Cockerell 1907

##### Notes

Table 1: Sites 1-4.

#### Dianthidium (Dianthidium) heterulkei

Schwarz 1940

##### Notes

New species record for Montana (Timberlake 1943, Grigarick and Stange 1968; Table 1: Site 3). The closest record reported in Hurd (1979) for this species is from neighbouring state Wyoming.

#### Dianthidium (Dianthidium) subparvum

Swenk 1914

##### Notes

Table 1: Sites 2, 3.

#### Stelis (Stelis) calliphorina

(Cockerell 1911)

##### Notes

Table 1: Site 3.

#### Stelis (Stelis) foederalis group sp. 6

##### Notes

Table 1: Site 3.

#### Stelis (Stelis) lateralis

Cresson 1864

##### Notes

Table 1: Sites 2, 4.

#### Stelis (Stelis) montana

Cresson 1864

##### Notes

Table 1: Site 3.

#### Stelis (Stelis) occidentalis

Parker & Griswold 2013

##### Notes

New species record for Montana (Parker and Griswold 2013; Table 1: Site 3). The closest records reported in Parker and Griswold (2013) for this species are from neighbouring Canadian province British Columbia and from neighbouring US state Idaho.

#### Stelis (Stelis) permaculata

Cockerell 1898

##### Notes

Table 1: Site 2.

#### Stelis (Stelis) subcaerulea

Cresson 1878

##### Notes

New species record for Montana (Cockerell 1911; Table 1: Site 3). The closest record reported in Hurd (1979) for this species is from neighbouring state Wyoming.

#### Stelis (Stelis) subemarginata group sp. 1

##### Notes

Table 1: Sites 2, 4.

#### Megachilini

#### Coelioxys (Boreocoelioxys) moestus

Cresson 1864

##### Notes

Table 1: Sites 2, 3.

#### Coelioxys (Boreocoelioxys) rufitarsis

Smith 1854

##### Notes

Table 1: Sites 1-4.

#### Coelioxys (Coelioxys) sodalis

Cresson 1878

##### Notes

Table 1: Site 4.

#### Coelioxys (Synocoelioxys) alternatus

Say 1837

##### Notes

Table 1: Sites 2, 3.

#### Megachile (Argyropile) parallela

Smith 1853

##### Notes

Table 1: Sites 1, 3, 4.

#### Megachile (Chelostomoides) angelarum

Cockerell 1902

##### Notes

Table 1: Sites 2, 3.

#### Megachile (Chelostomoides) campanulae

(Robertson 1903)

##### Notes

Table 1: Site 2.

#### Megachile (Eutricharaea) apicalis

Spinola 1808

##### Ecological interactions

###### Native status

Non-native to North America.

##### Notes

Table 1: Sites 2-4.

#### Megachile (Eutricharaea) rotundata

(Fabricius 1787)

##### Ecological interactions

###### Native status

Non-native to North America.

##### Notes

Table 1: Sites 1, 3, 4.

#### Megachile (Litomegachile) brevis

Say 1837

##### Notes

Table 1: Site 1.

#### Megachile (Megachile) lapponica

Thomson 1872

##### Notes

Table 1: Sites 2, 3.

#### Megachile (Megachile) montivaga

Cresson 1878

##### Notes

Table 1: Sites 2-4.

#### Megachile (Megachile) relativa

Cresson 1878

##### Notes

Table 1: Sites 1-4.

#### Megachile (Megachiloides) anograe

Cockerell 1908

##### Notes

Table 1: Site 4.

#### Megachile (Megachiloides) subnigra

Cresson 1879

##### Notes

Table 1: Site 1.

#### Megachile (Sayapis) pugnata

Say 1837

##### Notes

Table 1: Sites 1-4.

#### Megachile (Xanthosarus) frigida

Smith 1853

##### Notes

Table 1: Sites 1, 2, 4.

#### Megachile (Xanthosarus) latimanus

Say 1823

##### Notes

Table 1: Site 1.

#### Megachile (Xanthosarus) melanophaea

Smith 1853

##### Notes

Table 1: Sites 1-3.

#### Megachile (Xanthosarus) perihirta

Cockerell 1898

##### Notes

Table 1: Sites 1-4.

#### Osmiini

#### Ashmeadiella (Ashmeadiella) bucconis

(Say 1837)

##### Notes

Table 1: Sites 1, 3, 4.

#### Heriades (Neotrypetes) carinata

Cresson 1864

##### Notes

Table 1: Sites 1-4.

#### Heriades (Neotrypetes) cressoni

Michener 1938

##### Notes

Table 1: Sites 2, 3.

#### Heriades (Neotrypetes) variolosa

(Cresson 1872)

##### Notes

Table 1: Sites 1-4.

#### Hoplitis (Alcidamea) fulgida

(Cresson 1864)

##### Notes

Table 1: Sites 2, 3.

#### Hoplitis (Alcidamea) grinnelli

(Cockerell 1910)

##### Notes

Table 1: Sites 2-4.

#### Hoplitis (Alcidamea) hypocrita

(Cockerell 1906)

##### Notes

Table 1: Site 2.

#### Hoplitis (Alcidamea) pilosifrons

(Cresson 1864)

##### Notes

Table 1: Site 4.

#### Hoplitis (Alcidamea) producta

(Cresson 1864)

##### Notes

Table 1: Sites 1-4.

#### Hoplitis (Alcidamea) spoliata

(Provancher 1888)

##### Notes

New species record for Montana (Michener 1947, Mitchell 1960; Table 1: Sites 3, 4). Unpublished record on DiscoverLife (Suppl. material 2). The closest records reported in Michener (1947) for this species are from neighbouring Canadian provinces British Columbia and Saskatchewan and from neighbouring US state North Dakota.

#### Hoplitis (Formicapis) robusta

(Nylander 1848)

##### Notes

Table 1: Sites 2-4.

#### Osmia (Cephalosmia) marginipennis

Cresson 1878

##### Notes

Table 1: Site 2.

#### Osmia (Cephalosmia) subaustralis

Cockerell 1900

##### Notes

Table 1: Site 3.

#### Osmia (Helicosmia) coloradensis

Cresson 1878

##### Notes

Table 1: Sites 2-4.

#### Osmia (Melanosmia) aff. paradisica

Sandhouse 1924

##### Notes

Table 1: Sites 3, 4.

#### Osmia (Melanosmia) aff. phaceliae

Cockerell 1907

##### Notes

Table 1: Site 3.

#### Osmia (Melanosmia) albolateralis

Cockerell 1906

##### Notes

Table 1: Sites 2-4.

#### Osmia (Melanosmia) atrocyanea

Cockerell 1897

##### Notes

Table 1: Site 2.

#### Osmia (Melanosmia) brevis

Cresson 1864

##### Notes

Table 1: Sites 2, 3.

#### Osmia (Melanosmia) bruneri

Cockerell 1897

##### Notes

Table 1: Site 1.

#### Osmia (Melanosmia) ednae

Cockerell 1907

##### Notes

Table 1: Sites 2-4.

#### Osmia (Melanosmia) kincaidii

Cockerell 1897

##### Notes

Table 1: Site 3.

#### Osmia (Melanosmia) odontogaster group sp. 1

##### Notes

Formerly, subgenus Acanthosmioides Ashmead (Rightmyer et al. 2013; Table 1: Site 1).

#### Osmia (Melanosmia) paradisica

Sandhouse 1924

##### Notes

Table 1: Site 4.

#### Osmia (Melanosmia) pusilla

Cresson 1864

##### Notes

Table 1: Sites 2-4.

#### Osmia (Melanosmia) sculleni

Sandhouse 1939

##### Notes

Table 1: Site 4.

#### Osmia (Melanosmia) sedula

Sandhouse 1924

##### Notes

New species record for Montana (Donovan 1977, Sandhouse 1939, White 1952; Table 1: Sites 2-4). The closest records reported in Sandhouse (1939) and White (1952) for this species are from neighbouring Canadian province British Columbia and from neighbouring US state Wyoming.

#### Osmia (Melanosmia) simillima

Smith 1853

##### Notes

Table 1: Sites 2, 3.

#### Osmia (Melanosmia) tersula

Cockerell 1912

##### Notes

Table 1: Site 3.

#### Osmia (Melanosmia) trevoris

Cockerell 1897

##### Notes

Table 1: Sites 1-4.

#### Osmia (Melanosmia) tristella

Cockerell 1897

##### Notes

Table 1: Sites 2-4.

#### Osmia (Melanosmia) sp. F1

##### Notes

Table 1: Site 3.

## Analysis

Over 3 years (2013-2015), we collected 12,203 bees representing 202 species and morphospecies from 32 genera and five families; the list includes wild native, wild non-native and managed non-native bee species (reviewed in Russo 2016, see 'native status' in checklist, Suppl. materials 3, 4). We found that 25 species are new published records for the state of Montana (Suppl. material 5). The total number of specimens, genera and species for each family are as follows: Andrenidae: 1,510 specimens, 5 genera, 36 species and morphospecies; Apidae: 2,761 specimens, 10 genera, 49 species and morphospecies; Colletidae: 552 specimens, 2 genera, 18 species; Halictidae: 6,455 specimens, 6 genera, 35 species and morphospecies; Megachilidae: 925 specimens, 9 genera, 64 species and morphospecies. Analysis with EstimateS (Colwell 2013) yielded a Chao1 mean prediction of 251 bee species for the area (SD = 18.01; 95% confidence interval = 226.48-300.51 species), which is considerably higher than the observed number of species collected (Suppl. material 6).

## Discussion

Our study provides information on the wild bee species associated with diverse, small-scale agricultural farmlands in south-western Montana, expands the known distribution of several bee species to Montana and adds to a growing state list. The 25 new state records reported in this study brings the total number of published species records in the state to 399. Coupled with other recently published works (Dolan et al. 2017, Kuhlman and Burrows 2017, Reese et al. 2018), the high percentage (12.4%) of new state records amongst the bees at our sites further highlights the lack of published data and the need for additional bee biodiversity surveys throughout the state to develop a complete species list, especially considering that our study was conducted within an area measuring approximately 1,810 km^2^ (< 1% the size of the state). For example, a checklist of bees recently published reported 34 new Montana state records at a site ca. 260 km from ours (Kuhlman and Burrows 2017). Additionally, two new state records were recorded the same year for bumble bees (Dolan et al. 2017), one of the most well-known and easily recognisable groups amongst the wild bees. At least six unpublished species records are reported by Reese et al. (2018). Considering that two of these lists are from just one county each (present study and Kuhlman and Burrows 2017) and one (Reese et al. 2018) included three of Montana’s 56 counties and that Montana comprises a large, topographically and ecologically diverse region with disparate climatic conditions, this is only a start to the additional surveying that needs to be conducted across the state.

It is not currently possible to accurately compare the number of bee species in Montana to other US states and Canadian provinces, since we are far from a statewide inventory. In addition, few species lists have been published for western US states, though lists have been published for several mid-western and north-eastern US states. The closest, comprehensive, statewide bee list is from Colorado which has 946 species (Scott et al. 2011). Wyoming, Montana’s neighbour to the south, has 487 documented bee species with additional species (>150) predicted based on distributional patterns, though faunal assessments are not complete (Lavigne and Tepedino 1976). To the north of Montana in Canada, so far, ca. 225 bee species have been recorded in Saskatchewan, ca. 325 species in Alberta and ca. 425 species in British Columbia (Canadian Endangered Species Conservation Council 2016).

Many of the bee species we document as new state records have distributions predicted to include Montana or records from states and provinces that either border Montana or are reasonably close to Montana (and with similar ecosystems). We highlight two bee species on this checklist, *L. clematisellum* and *M. niveus*, whose known distributions are considerably increased with their documentation in Montana. The south-western US distribution of *L. clematisellum* previously included New Mexico, Arizona, California and Utah (Sandhouse 1924, Hurd 1979); unpublished records exist also in Wyoming (pers. comm. Joel Gardner). The eastern US distribution of *M. niveus* ranged from New York to Minnesota and Kansas, south to North Carolina, Alabama and Mississippi (LaBerge 1961). Our findings support the importance of documenting Montana’s bee fauna for understanding the full distributional ranges of the wild bees in North America and for producing more comprehensive regional keys.

In addition to wild, native bee species, we also documented four non-native species, including two economically important, commercially-managed species intentionally introduced for crop pollination and two wild, non-managed species accidentally introduced to the US (Klein et al. 2007, Pitts-Singer and Cane 2011, Russo 2016). Managed honey bee, *Apis mellifera*, colonies were located at one of our sites, as well as nearby sites and were captured in bowl traps and observed visiting our experimental crop strips and wildflower strips. We also captured alfalfa leafcutting bees, *Megachile rotundata*, in bowl traps and with nets from two non-native plant species, *Lotus corniculatus* L. and *Melilotus officinalis* (L.) Lam. *Megachile rotundata* are managed for alfalfa (*Medicago sativa* L.) seed production (Pitts-Singer and Cane 2011 in Montana, an important alfalfa seed-producing state (USDA-NASS 2017). Another leafcutting bee *Megachile apicalis* was recently documented in the literature to occur in Montana (Kuhlman and Burrows 2017) and was netted from *E. speciosus* in our wildflower strips as well as captured in bowls. Last, the yellow-faced bee *Hylaeus leptocephalus* was netted from *E. speciosus* and *G. viscosissimum* in our flower strips. The impacts (positive and negative) of both intentionally and accidentally introduced non-natives on other bee species (e.g. competition for floral resources and nesting sites and pathogen transmission) and plants (e.g. pollination of native plants, invasive weeds and agricultural crops) require further study (reviewed in Russo 2016).

Our results underestimate the actual bee richness from this study. Though the vast majority of bees were identified to species in our study, the absence of revisionary studies for several genera or subgenera precluded morphospecies sorts for some and, for others, the morphospecies counts may be low because male morphospecies, some of which might not be conspecific with any of the female morphospecies, were not counted. Bees in one genus, *Nomada*, could only be designated as morphospecies and bee species of *Sphecodes* and Lasioglossum (subgenus Evylaeus) were classified only to the generic or subgeneric level. In addition, only a fraction of the Lasioglossum in the subgenus Dialictus, which accounted for about 25% of the specimens collected in our study, were identified to species. Bees in the subgenus Dialictus are very abundant in studies using bowl traps (Droege et al. 2010) and the lack of species-level identifications for this group are similar amongst faunistic studies in Montana (e.g. over 22,000 unidentified *Dialictus* in Kuhlman and Burrows 2017 and over 2,500 unidentified *Dialictus* in Adhikari et al. In prep). All of these groups require taxonomic work in the western US before species can be fully resolved and will likely contribute dozens of species to a state list for Montana once identified.

Different habitats throughout the state, particularly farmlands versus wildland habitats, are likely to support different suites of bee species. For example, between 33-43% of species were unique to our study when compared to all species (excluding morphospecies) documented in each of two studies conducted in montane wildland habitats in Montana (Kuhlman and Burrows 2017, Reese et al. 2018). However, between 57-66% of bee species in our study were shared with each of those same two studies, indicating considerable overlap with our study and that some bee species can be supported by both types of habitats. When we compared bee species (excluding morphospecies) from all three studies, we found 58 of 195 species (30%) unique to Kuhlman and Burrows (2017), 73 of 226 species (32%) unique to Reese et al. (2018) and 40 of 170 species (24%) unique to our study. All three studies shared 81 species in common, accounting for almost half (48%) of the species in our study, 42% of the species in Kuhlman and Burrows (2017) and 36% in Reese et al. (2018). However, our comparisons did not account for differences in collection methods, sampling effort and geographic area amongst studies, all important determinants of species overlap. More standardised inventories, as well as complete state-wide surveys, are needed for more accurate comparisons between studies and habitats. (Such efforts are hampered by the taxonomic impediment.)

In contrast, when we compared our study to another conducted in a highly-simplified, small grains-wheat farming system in north central (Chouteau County) Montana (i.e. the drylands of the Northern Great Plains), we found 73% of bee species were unique to our study compared to those reported by Adhikari et al. (In prep), 27% of bee species were shared (four species are amongst the new state records reported here) and 39 of 85 species (46%) of bee species were unique to Adhikari et al. (In prep). These results suggest the bee communities, which these two agricultural habitats support, are quite different and may be further driven by regional ecosystem diversity. Furthermore, if we compare all four studies, only 28 bee species (16% of the bees in our study) are shared. Again, methodological differences (e.g. sampling intensity, geographic area) between studies make comparisons difficult. Additional surveying is greatly needed in different habitats throughout the state to better understand the basic biology, ecology and distribution of Montana’s wild bees, whose importance to natural- and agroecosystems is not fully understood. These types of data are valuable for directing projects aimed at supporting farmland biodiversity and for conserving wild, native bees in general throughout the state.

## Supplementary Material

Supplementary material 1 Supplementary Table 1 Data type: weather data
File: oo_230944.docx C. M. Delphia, T. Griswold, E. G. Reese, K. M. O'Neill, L. A. Burkle

Supplementary material 2 Supplementary Table 2 Data type: occurence
File: oo_230945.xlsx C. M. Delphia, T. Griswold, E. G. Reese, K. M. O'Neill, L. A. Burkle

Supplementary material 3 Supplementary Table 3 Data type: checklist
File: oo_253349.xls C. M. Delphia, T. Griswold, E. G. Reese, K. M. O'Neill, L. A. Burkle

Supplementary material 4 Supplementary Table 4 Data type: checklist
File: oo_253350.xlsx C. M. Delphia, T. Griswold, E. G. Reese, K. M. O'Neill, L. A. Burkle

Supplementary material 5 Supplementary Table 5 Data type: occurence
File: oo_247377.xlsx C. M. Delphia, T. Griswold, E. G. Reese, K. M. O'Neill, L. A. Burkle

Supplementary material 6 Supplementary Table 6 Data type: species
Brief description: Raw data for Chao1 analysis.
File: oo_247378.xlsx C. M. Delphia, T. Griswold, E. G. Reese, K. M. O'Neill, L. A. Burkle

## Figures and Tables

**Table 1.** Site number, site name, latitude, longitude and elevation of farms sampled in 2013-2015 within a 24 km radius of Bozeman, Montana in Gallatin County.

**Site Number**	**Study Site**	**Latitude**	**Longitude**	**Elevation (m)**
1	Gallatin Grown	N45°46.28’	W111°17.72’	1350
2	Gallatin Valley Botanical	N45°39.63’	W110°56.85’	1511
3	Rocky Creek Farm	N45°39.82’	W110°56.95’	1508
4	Towne’s Harvest Garden	N45°39.92’	W111°04.35’	1490

**Table 2.** List of published keys used for species identification. Genus-level identifications were done using Michener et al. (1994).

**Family**	**References**
Andrenidae	Bouseman and LaBerge 1979, Donovan 1977, LaBerge 1967, LaBerge 1969, LaBerge 1973, LaBerge 1977, LaBerge 1980, LaBerge 1985, LaBerge 1986, LaBerge 1989, LaBerge and Bouseman 1970, LaBerge and Ribble 1975, Ribble 1967, Ribble 1974, Thorp 1969, Timberlake 1956, Timberlake 1960, Timberlake 1964, Timberlake 1967
Apidae	Brumley 1965, Daly 1973, Hurd and Linsley 1951, Koch et al. 2012, LaBerge 1956b, LaBerge 1956a, LaBerge 1961, LaBerge 1963, Rightmyer 2008, Sipes 2001, Thorp et al. 1983, Williams et al. 2014
Colletidae	Snelling 1966b, Snelling 1966a, Snelling 1970, Stephen 1954
Halictidae	Coelho 2004, Dumesh and Sheffield 2012, Gibbs 2010, McGinley 1986, McGinley 2003, Roberts 1972, Roberts 1973
Megachilidae	Baker 1975, De Silva and Packer 2012, Gonzalez and Griswold 2013, Grigarick and Stange 1968, Hurd and Michener 1955, Michener 1938, Michener 1939, Michener 1947, Rightmyer et al. 2010, Sandhouse 1939, Sheffield et al. 2011
