# Supplementary material for: Checklist of bees (Hymenoptera: Apoidea) from small diversified vegetable farms in south-western Montana
Source: Biodivers Data J. 2019 Jan 28;(7):e30062. doi: 10.3897/BDJ.7.e30062 (PMC6361878; doi:10.3897/BDJ.7.e30062)
Supplement: Supplementary material 1 — Supplementary Table 1 [file bdj-07-e30062-s001.docx]

**Supplementary Table 1.** Mean minimum and maximum temperature and precipitation values for Bozeman, Montana; period of record from 08April1892 to09June2016 (Western Regional Climate Center 2018).

|  | **Jan** | **Feb** | **Mar** | **Apr** | **May** | **Jun** | **Jul** | **Aug** | **Sep** | **Oct** | **Nov** | **Dec** | **Annual** |
| --- | --- | --- | --- | --- | --- | --- | --- | --- | --- | --- | --- | --- | --- |
| Max temperature (°C) | -0.17 | 1.94 | 5.94 | 12.17 | 17.22 | 22.00 | 27.45 | 26.84 | 20.78 | 14.22 | 5.67 | 0.89 | 12.89 |
| Min temperature (°C) | -11.11 | -9.28 | -5.89 | -0.89 | 3.56 | 7.33 | 10.61 | 9.72 | 5.11 | 0.50 | -5.44 | -9.72 | -0.44 |
| Precipitation (mm) | 22.10 | 18.54 | 34.04 | 48.01 | 73.41 | 73.91 | 34.29 | 31.50 | 43.18 | 39.12 | 28.45 | 22.35 | 469.39 |

Original temperature values were reported in °F and precipitation in inches.
